# Effects of Process Variables on Properties of CoFe_2_O_4_ Nanoparticles Prepared by Solvothermal Process

**DOI:** 10.3390/nano11113056

**Published:** 2021-11-13

**Authors:** Hong Diu Thi Duong, Dung The Nguyen, Kyo-Seon Kim

**Affiliations:** 1 Department of Chemical Engineering, Kangwon National University, Chuncheon 200-701, Kangwon-do, Korea; duonghongdiu97chy@gmail.com; 2 Faculty of Chemistry, University of Sciences, Vietnam National University, 19 Le Thanh Tong, Hoan Kiem, Hanoi 100000, Vietnam; nguyentd@hus.edu.vn; 3 CIRI University-Industry Cooperation Laboratory, University of Ulsan, Ulsan 44776, Korea

**Keywords:** cobalt ferrite nanoparticles, controlled synthesis, solvothermal method, oleic acid, magnetic properties

## Abstract

Controlling the morphology and magnetic properties of CoFe_2_O_4_ nanoparticles is crucial for the synthesis of compatible materials for different applications. CoFe_2_O_4_ nanoparticles were synthesized by a solvothermal method using cobalt nitrate, iron nitrate as precursors, and oleic acid as a surfactant. The formation of CoFe_2_O_4_ nanoparticles was systematically observed by adjusting synthesis process conditions including reaction temperature, reaction time, and oleic acid concentration. Nearly spherical, monodispersed CoFe_2_O_4_ nanoparticles were formed by changing the reaction time and reaction temperature. The oleic acid-coated CoFe_2_O_4_ nanoparticles inhibited the growth of particle size after 1 h and, therefore, the particle size of CoFe_2_O_4_ nanoparticles did not change significantly as the reaction time increased. Both without and with low oleic acid concentration, the large-sized cubic CoFe_2_O_4_ nanoparticles showing ferromagnetic behavior were synthesized, while the small-sized CoFe_2_O_4_ nanoparticles with superparamagnetic properties were obtained for the oleic acid concentration higher than 0.1 M. This study will become a basis for further research in the future to prepare the high-functional CoFe_2_O_4_ magnetic nanoparticles by a solvothermal process, which can be applied to bio-separation, biosensors, drug delivery, magnetic hyperthermia, etc.

## 1. Introduction

Magnetic nanoparticles are very attractive for many applications in various fields, among which the iron oxide (Fe_3_O_4_) nanoparticles have been widely studied in the past decade due to their outstanding ability to capture the magnetic moment signal, high biocompatibility, and high chemical stability [1,2]. However, currently, magnetic ferrite nanoparticles have other transition metal atoms such as Ni, Cu, Mg, Zn, Co, and Mn instead of some iron atoms in the ferrite crystal lattice and have gained remarkable attention in recent years because of their improved unique physicochemical properties such as a high surface area-to-volume ratio, feasibility of surface functionalization, and excellent magnetic responses with magnetic fields and field gradients that can be widely applied to bio-separation, magnetic resonance imaging, biosensors, drug delivery, and magnetic hyperthermia [3,4,5,6,7,8,9,10]. The spinel-type ferrite nanoparticles (MFe_2_O_4_, where M(II) is a d-block transition metal such as Zn, Co, Mn, etc.) displayed remarkably enhanced properties. For example, Jang et al. [11] varied the amounts of Zn and Mn in the ferrite nanoparticles and found that the (Zn*_x_*M_1-*x*_)Fe_2_O_4_ ((M = Mn^2+^, Fe^2+^) nanoparticles exhibited much higher magnetism than conventional Fe_3_O_4_ nanoparticles, which, consequently, led to 8 to 14 times greater r2 (MRI contrast effect) values for magnetic resonance imaging and 4 times greater specific loss power (SLP) values for hyperthermia cancer cell treatments than conventional magnetic nanoparticles. The MFe_2_O_4_ nanomaterials with specific physicochemical and magnetic properties have been synthesized over the years and have been conquering new horizons in numerous research fields, including high-density magnetic storage, catalysis, and biomedical theranostics. Among the MFe_2_O_4_ nanomaterials, CoFe_2_O_4_ nanoparticles are of great interest for biomedical applications because of their highest saturation magnetization and the highest SLP level compared to magnetite and manganese ferrite nanoparticles [12,13].

It has been widely reported that particle size, shape, composition, and structural defects are important factors that strongly influence the magnetic behaviors and, consequently, applications of the CoFe_2_O_4_ nanoparticles [14,15]. For example, the ferromagnetic CoFe_2_O_4_ nanoparticles have the advantages for permanent magnet applications such as magnetic recording and energy storage [16], while the superparamagnetic CoFe_2_O_4_ nanoparticles have the merits for biomedical applications such as hyperthermia treatment, drug delivery, and cancer therapy [17]. The CoFe_2_O_4_ nanoparticles could be synthesized by various methods including sol-gel combustion [18], thermal decomposition [19], co-precipitation [5], microemulsion [20], and solvothermal [21] and polyol [22] approaches. It should be emphasized that different synthesis methods or different synthesis process variables might cause large differences in the resulting magnetic properties of ferrite nanomaterials. The co-precipitation method has been known as the most convenient method to synthesize a large amount of ferrite nanoparticles at either room temperature or elevated temperature, but the synthesized nanoparticles usually exhibit a low degree of crystallinity and large polydispersity [23]. The microemulsion method has been more useful to obtain a narrower size range and more uniform physical properties of ferrite nanoparticles. However, this method generally involves the complicated steps to generate a uniform and stable emulsion system for further formation of the ferrite nanoparticles. In addition, the yield of product is relatively low and, thus, this method is not a very efficient process for scale-up [24]. The solvothermal methods have become popular and widely used to synthesize ferrite nanomaterials due to their simplicity, low cost, high potential on a large-scale fabrication, and, more importantly, high uniformity in both size and shape with excellent magnetic properties of the synthesized nanoparticles [25]. Since the chemical reactions take place in a closed one-pot system at relatively high temperature and high pressure, all process variables must be well designed and set up in advance. For example, ferromagnetic CoFe_2_O_4_ spheres with porous/hollow nanostructures were successfully synthesized through solvothermal processes [26]. The formation of such porous/hollow structures during the solvothermal processes involved the burst formation of small-size ferrite nanoparticles, subsequently assembly formation of those small-size nanoparticles, and, finally, particle growth via the Ostwald ripening process [27]. By introducing a strong surfactant like oleic acid, Jovanović et al. [28] reported that oleic acid formed the covalent bidentate with metal ions on the particle surface and a complete monolayer was formed at the critical concentration, which controlled the particle nucleation, growth, and assembly and eventually resulted in the formation of specific nanoparticle products with different sizes and shapes. Munjal et al. [14] also utilized oleic acid as a surfactant to synthesize monodispersed oleic-coated CoFe_2_O_4_ nanoparticles with high uniformity of both particle size and shape. The CoFe_2_O_4_ nanoparticles exhibited superparamagnetic characteristics due to the small-size effect and, thus, would be suitable for hyperthermia treatment. Repko et al. [29] reported that the nucleation and growth of CoFe_2_O_4_ nanoparticles could be terminated by controlling the solvent of pentanol or ethanol in the precursor solution, which facilitated the formation of smaller nanoparticles with better size distribution.

It is still a great challenge to controllably synthesize the CoFe_2_O_4_ nanoparticles of desired size, shape, and properties for proposed application, because a small difference in synthesis process’ conditions might eventually cause a remarkable variation of product particle morphologies and characteristics. This will require a comprehensive study about the effects of synthesis process variables on the products. In this study, we synthesized CoFe_2_O_4_ nanoparticles in a solution system containing oleic acid, water, and ethanol by a solvothermal process. The effects of major process variables such as reaction temperature, reaction time, and oleic acid concentration on the morphologies and characteristics of CoFe_2_O_4_ nanoparticles were systematically investigated. We strongly believe that this study can be considered as a valuable protocol for the synthesis of morphology-controlled CoFe_2_O_4_ nanoparticles, because the particle morphology control is strictly required to synthesize highly uniform products with desired properties for proposed applications.

## 2. Materials and Methods

### 2.1. Chemicals

Deionized (DI) water was used to prepare all aqueous solutions. All chemicals used for this study are as follows: iron (III) nitrate nonahydrate (Fe(NO_3_)_3_·9H_2_O, ≥99%) (Daejung, Gyeonggi, Korea), cobalt (II) nitrate hexahydrate (Co(NO_3_)_2_·6H_2_O, Daejung, ≥98%), sodium hydroxide (NaOH, ≥96%)(Yakuri, Kyoto, Japan), ethanol (C_2_H_6_O, Daejung, ≥99.9%), 1- pentanol (C_5_H_12_O, ≥99%)(Junsei, Tokyo, Japan), n- hexane (C_6_H_14_, Daejung, ≥95%), and oleic acid (C_18_H_34_O_2_, Daejung, extra pure).

### 2.2. Synthesis of Cobalt Ferrite Nanoparticles

The CoFe_2_O_4_ was synthesized solvothermally by conducting the reaction of metal oleate complexes in a mixture solution containing oleic acid, water, and ethanol. Firstly, a mixture of metal (Co^2+^, Fe^3+^)–oleate complexes was prepared priorly by reactions of iron nitrate, cobalt nitrate with sodium hydroxide, and oleic acid in ethanol. Specifically, 10 mmol NaOH was dissolved in 2 mL distilled water and 10 mL ethanol was added, followed by a drop-by-drop addition of 3.8 mL oleic acid. The solution was vigorously stirred for 15 min and then was transferred to a Teflon autoclave cell. Another solution was prepared by dissolving 2 mmol Fe(NO_3_)_3_·9H_2_O and 1 mmol Co(NO_3_)_2_·6H_2_O in 18 mL DI water and stirring for 15 min and then was added drop by drop into the solution in a Teflon autoclave cell, which was stirred for 2 h by a magnetic stirrer afterwards. The autoclave cell with prepared solution was placed into an oven with controlled temperatures (120 °C, 140 °C, 160 °C, 180 °C, and 200 °C) for different processing times (1 h, 2 h, 4 h, 8 h, 12 h, and 16 h). Tap water was used to quickly cool down the autoclave cell to room temperature. The sediment product containing CoFe_2_O_4_ nanoparticles was collected by a permanent magnet, washed with hexane and then by ethanol four times. Finally, the CoFe_2_O_4_ nanoparticles were dried at 60 °C for 6 h before further characterization. The role of oleic acid was systematically investigated by varying its concentration from 0 to 1.5 M while keeping the same conditions for the other experimental variables.

### 2.3. Characterization

The crystal structure of the CoFe_2_O_4_ nanoparticles was analyzed by powder X-ray diffraction (XRD) using a X’Pert–PRO (PANalytical, Almelo, The Netherlands) diffractometer with Cu-Kα radiation (λ = 1.5406 Å) and a scanning speed of 10°/s in the *2θ* range of 10°–80°. The average crystallite size (dXRD) was calculated from the full width at the half maximum of (311) peak by using the Scherrer formula [25]:

(1) $$d=\frac{K\lambda }{\beta cos\theta }$$

where *d, K, λ, β*, and *θ* are the average crystalline size (nm), Scherrer constant, which has a value of 0.9, wavelength (nm), full width at half maximum of diffraction peaks, and diffraction angle (Brag’s angle), respectively.

The morphology of CoFe_2_O_4_ nanoparticles was examined by using a JEM-2100F (Tokyo, Japan) transmission electron microscope TEM. The CoFe_2_O_4_ nanoparticles were first dispersed in n-hexane and washed by ultrasonic treatment for 30 min. Then, 0.01 wt% CoFe_2_O_4_ nanoparticle solution dispersed in hexane was dropped on a carbon-coated copper grid and then dried for 6 h before TEM measurement. X-ray spectroscopy (EDS) was utilized to determine the elemental composition of samples by using a S-4800 Field Emission Scanning Electron Microscope FE-SEM instrument (Hitachi, Tokyo, Japan).

Fourier-transform infrared (FTIR) spectroscopy and thermogravimetric analysis (TGA) were used to characterize the absorption of oleic acid on the surface of CoFe_2_O_4_ nanoparticles quantitatively. The FTIR spectra were recorded in the wavelength range of 500–4000 cm^−1^ by using Spectrum GX (Perkin Elmer, Waltham, MA, USA) equipped with the Frontier model. The TGA analysis was carried out by using the SDT Q600 instrument (TA instruments, DE, USA) with a heating rate of 10 °C/min in nitrogen gas for the temperature range of 25 °C–500 °C. The measured weight loss by TGA analysis was used to calculate the number of adsorbed oleic acid molecules (N) per unit surface area of CoFe_2_O_4_ nanoparticles by the following equation:

(2) $$N=\frac{2\rho dwN_{A}}{3M\left(100-w\right)}$$

where *ρ, d, w, N_A,_* and *M* are the density of CoFe_2_O_4_ nanoparticles (=5.23 g/cm^3^ [30]), average diameter of CoFe_2_O_4_ nanoparticles measured from TEM analysis, weight loss obtained from TGA results, which reflects the mass of oleic acid, Avogadro constant (=6.023 × 10^23^), and molecular weight of oleic acid (=282.47 g/mol), respectively, and (100 − *w*) reflects the mass of pure CoFe_2_O_4_ nanoparticles.

A PPMS-14 vibrating sample magnetometer (Quantum Design, San Diego, USA) was used for magnetization measurements with an applied magnetic field in the range of −15 to 15 kOe. The cation distribution of CoFe_2_O_4_ nanoparticles was characterized by LabRam Aramis laser Raman spectroscopy (Horiba Jobin Yvon, Irvine, USA) with a laser (533 nm) excitation source over the wavenumber range of 800–100 cm^−1^. Cobalt ferrite is a spinel oxide with the chemical composition of (Co_δ_^2+^Fe_1−δ_^3+^)_A_(Co_1−δ_^2+^ Fe_1+δ_^3+^)_B_O_4_, where A is the tetrahedral site, B is the octahedral site, and δ is the cation distribution factor, which presents the fraction of tetrahedral (A) site [31]. The Co content in the tetrahedral site was calculated by using the following equation [28,32]:

(3) $$\delta_{Raman}=\frac{I_{Co}}{2(I_{Co}+RI_{Fe})}$$

where *I_Co_* and *I_Fe_* are the intensities of A_1g_ (1) (~680 cm^−1^) and of A_1g_ (2) (~615 cm^−1^). A_1g_(1) and A_1g_ (2) demonstrated the stretching vibrations of the Fe–O and Co–O bonds in the tetrahedral site. Jovanović et al. [28] proposed that the R value of 0.5 can be applied for the oscillator strength of the Co–O bonds to the Fe–O bonds in the tetrahedral site of CoFe_2_O_4_ nanoparticles. In this study, we used this R value for the calculation of δ_Raman_ of our materials (CoFe_2_O_4_ nanoparticles) based on these previous works. The δ is special for the degree of inversion describing the cation distribution in the ferrite spinel structure.

## 3. Results

### 3.1. Effect of Reaction Time on the Morphology of CoFe_2_O_4_ Nanoparticles

**Figure 1:**
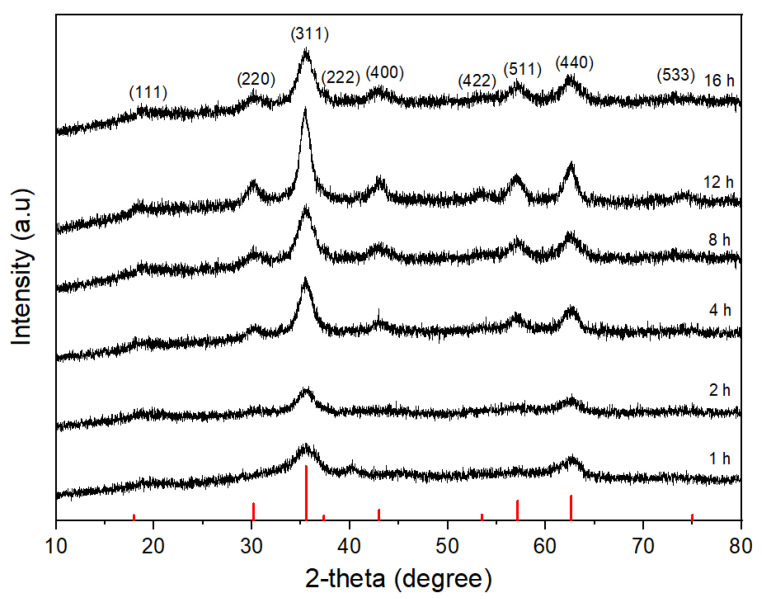
XRD patterns of CoFe_2_O_4_ nanoparticles synthesized at 180 °C in the presence of 1 M oleic acid for different reaction times.

**Figure 2:**
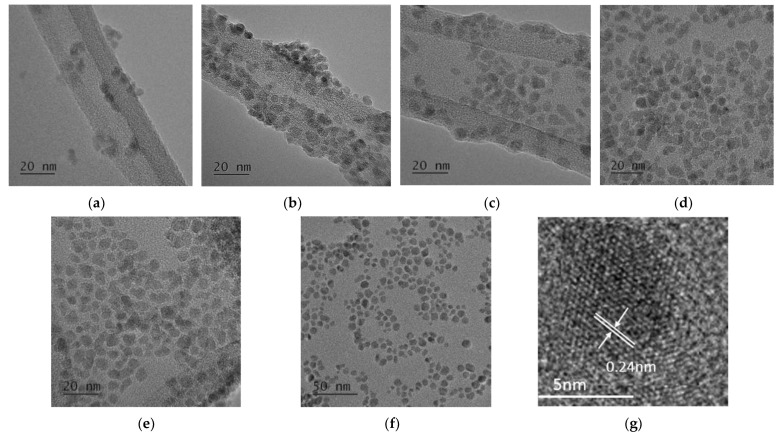
TEM images of CoFe_2_O_4_ nanoparticles synthesized at 180 °C in the presence of 1 M oleic acid for different reaction times of 1 h (**a**), 2 h (**b**), 4 h (**c**), 8 h (**d**), 12 h (**e**), and 16 h (**f**), and HR-TEM image of samples prepared for 16 h (**g**).

**Figure 3:**
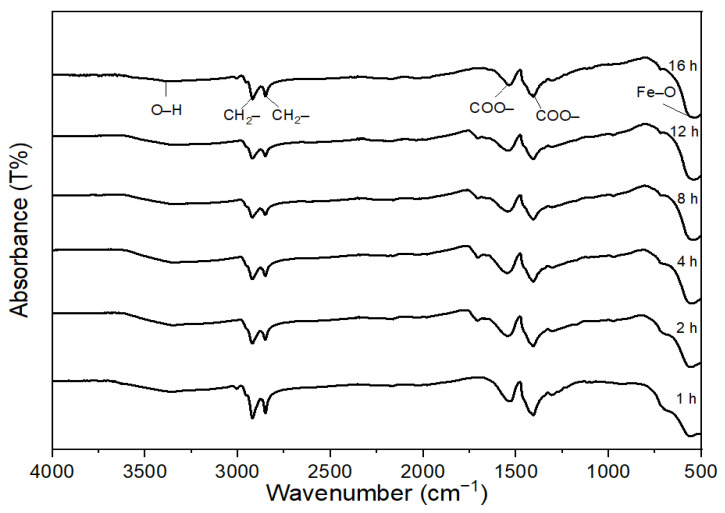
FT-IR spectra of CoFe_2_O_4_ nanoparticles synthesized at 180 °C in the presence of 1 M oleic acid for different reaction times.

Figure 1 shows the XRD patterns of CoFe_2_O_4_ nanoparticles prepared at 180 °C for different reaction times in an autoclave cell with a constant oleic acid concentration of 1 M. The diffraction peaks at *2θ* = 30.22°, 35.45°, 43.16°, 53.2°, 57.12°, 62.67°, and 74.3° correspond to (111), (220), (311), (400), (422), (511), (440), and (533) planes, respectively, of the cubic spinel structure of CoFe_2_O_4_ nanoparticles (JCPDS card no. 22-1086). The CoFe_2_O_4_ crystals started to form after 1 h with the most notable growth of the diffraction peaks at *2θ* = 35.45° and 62.67°, corresponding to the (311) and (440) planes, respectively. As the reaction time increased, the diffraction peaks corresponding to the (220), (400), (422), (511), and (533) planes also appeared. As the reaction time increased to 8–16 h, the intensity of those peaks increased remarkably, which means that the crystallinity of CoFe_2_O_4_ nanoparticles also increased. The average crystallite sizes were 4 nm, 4.2 nm, 4.3 nm, 5 nm, 5.6 nm, 5.8 nm, and 6 nm for the reaction time of 1 h, 2 h, 4 h, 8 h, 12 h, and 16 h, respectively, using the Scherrer formula. Figure 2 illustrates the TEM images of CoFe_2_O_4_ nanoparticles prepared at 180 °C for different reaction times. It could be clearly seen that well-separated nanoparticles with average particle sizes of 4 nm, 4.5 nm, 5 nm, 5.5 nm, 6.5 nm, and 7 nm were obtained for the reaction times of 1 h, 2 h, 4 h, 8 h, 12 h, and 16 h, respectively. The fringes in the CoFe_2_O_4_ nanoparticles were confirmed by HR-TEM. Figure 2g shows the lattice fringe with the fringe distance in single nanoparticles of 0.25 nm, which corresponds to the lattice spacing of (311) planes at 0.25 nm in the cubic spinel CoFe_2_O_4_. The oleic acid coverage was formed on the CoFe_2_O_4_ nanoparticles after 1 h of reaction time from FTIR results (Figure 3), but the oleic acid layer was not covering the total surface of CoFe_2_O_4_ nanoparticles and could not completely prevent the mass transfer to the CoFe_2_O_4_ nanoparticles. Therefore, the particle size of CoFe_2_O_4_ nanoparticles increased slightly as the reaction time increased. Table 1 shows the percentage elemental composition of the sample prepared at 180 °C for 16 h. It confirmed the presence of iron, cobalt, oxygen, and no impurities.

FT-IR spectra were measured for the CoFe_2_O_4_ nanoparticles obtained with different reaction times, as shown in Figure 3, to investigate the effect of oleic acid coated onto the surface of nanoparticles. The strong peak at 585.2 cm^−1^ corresponded to the vibration of the Fe–O bond from the octahedral site [33,34]. This frequency band in the FT-IR spectra of all samples was associated with the characteristic peaks of the CoFe_2_O_4_ spinel structure [35]. The presence of oleic acid was confirmed by asymmetric and symmetric –CH_2_ stretching at 2919.52 cm^−1^ and 2850 cm^−1^, respectively. Two other bands, at 1530.4 cm^−1^ and 1406.1 cm^−1,^ could be attributed to the asymmetric and symmetric stretching vibration of the COO– group from oleic acid, respectively [36]. The peak at 3368.55 cm^−1^ was attributed to the stretching vibration of –OH, which may have been from the presence of water in the samples. The peaks at the respective wavelengths are shown in Table 2. It was observed that oleic acid was adsorbed on the surface of the CoFe_2_O_4_ nanoparticles via its carboxylate group for all the reaction times (1–16 h). Because most of the CoFe_2_O_4_ nanoparticle surfaces were covered by oleic acid from the beginning of the particle growth in the autoclave cell, the mass transfer rate of precursors from solution to nanoparticles was limited, the particle growth rate was not fast, and the CoFe_2_O_4_ nanoparticle size increased slowly with the increase of reaction time, as confirmed by the TEM analysis in Figure 2. It should be emphasized that oleic acid played an important role as a surfactant to control the particle size in the autoclave cell for different reaction times.

**Figure 4:**
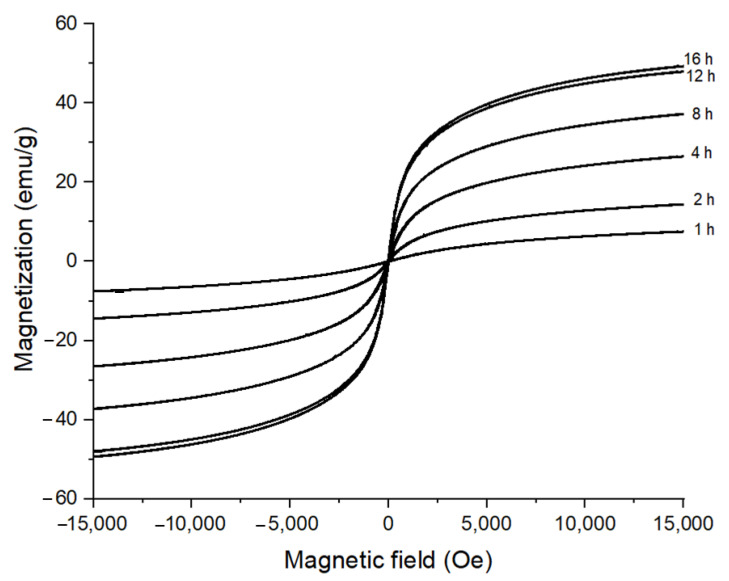
Room temperature M vs. H dependence of CoFe_2_O_4_ nanoparticles synthesized at 180 °C in the presence of 1 M oleic acid for different reaction times.

The magnetic properties of CoFe_2_O_4_ nanoparticles prepared at 180 °C with 1 M oleic acid for different reaction times are presented in Figure 4. The M versus H dependence confirmed the superparamagnetic behavior of the prepared CoFe_2_O_4_ nanoparticles. The magnetization values (M_S_) were 7.55 emu/g, 14.42 emu/g, 26.53 emu/g, 37.23 emu/g, 47.98 emu/g, and 49.35 emu/g for the reaction times of 1 h, 2 h, 4 h, 8 h, 12 h, and 16 h, respectively. The magnetization value of the CoFe_2_O_4_ nanoparticles increased quickly during the reaction time of 2–12 h, and, at 15,000 Oe of the magnetic field, increased from 7.55 emu/g to 49.35 emu/g (almost 6.5 times) with the increase of reaction time from 1 h to 16 h. This remarkable increase in the magnetic behavior of those CoFe_2_O_4_ nanoparticles was caused by the fast increases of crystallinity as well as of the particle size during the reaction time, as shown in Figure 1 and Figure 2 [37].

### 3.2. Effect of Reaction Temperature on the Morphology of CoFe_2_O_4_ Nanoparticles

**Figure 5:**
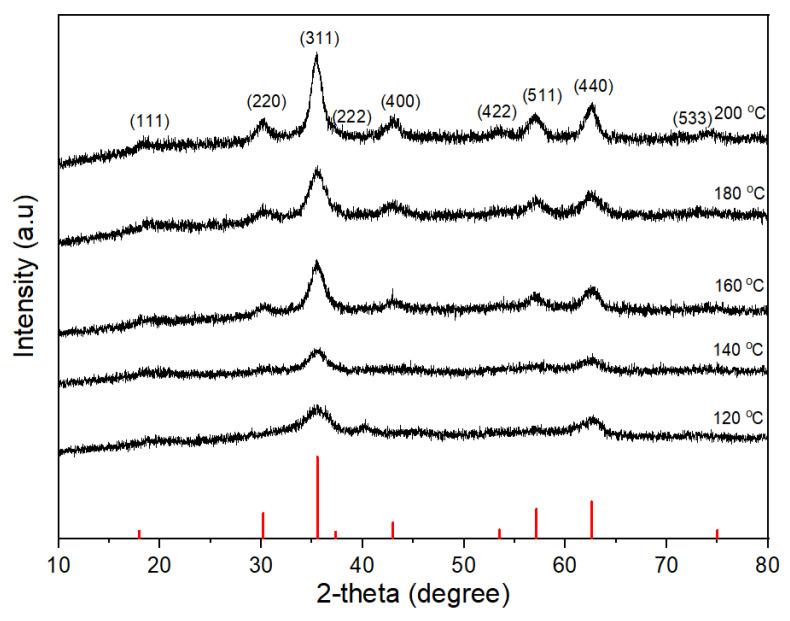
XRD patterns of CoFe_2_O_4_ nanoparticles synthesized at 16 h in the presence of 1 M oleic acid for different reaction temperatures.

**Figure 6:**
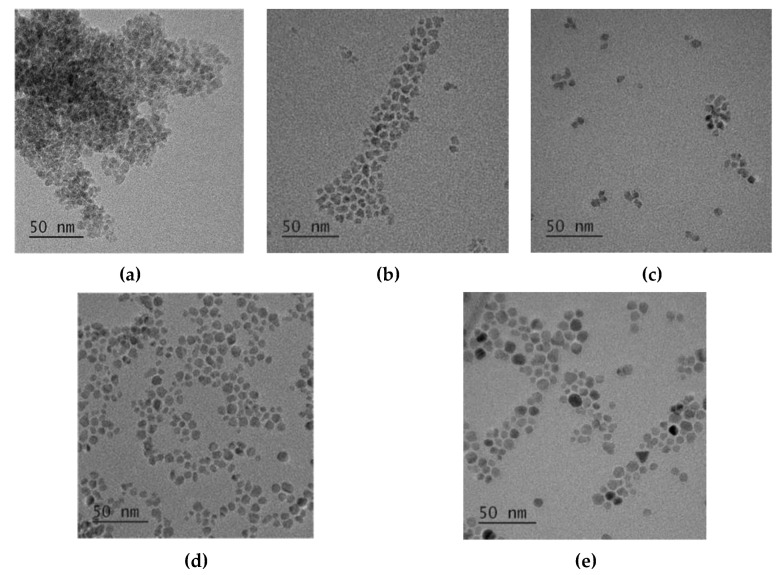
TEM images of cobalt ferrite nanoparticles synthesized at 16 h in the presence of 1 M oleic acid for different reaction temperatures 120 °C (**a**), 140 °C (**b**), 160 °C (**c**), 180 °C (**d**), 200 °C (**e**).

Figure 5 shows the XRD patterns of CoFe_2_O_4_ nanoparticles prepared at different reaction temperatures with 1 M oleic acid for 16 h. The cubic spinel structure of CoFe_2_O_4_ nanoparticles was confirmed for all reaction temperatures. All the diffraction peaks became sharper as the reaction temperature increased, which shows that the crystallinity of CoFe_2_O_4_ nanoparticles increased as the reaction temperature increased. The average crystallite sizes were about 4.5 nm, 4.9 nm, 5.4 nm, 5.8 nm, 6 nm, and 9.3 nm by the Scherrer formula for the reaction temperatures of 120 °C, 140 °C, 160 °C, 180 °C, and 200 °C, respectively. The TEM images of the CoFe_2_O_4_ nanoparticles synthesized for different reaction temperatures (Figure 6) revealed that the agglomerated CoFe_2_O_4_ nanoparticles were prepared at the reaction temperature of 120 °C, while the well-separated CoFe_2_O_4_ nanoparticles were prepared at the reaction temperature of 140 °C. The average particle sizes of 5.5 nm, 6.3 nm, 7 nm, and 12 nm were obtained for the reaction temperatures of 140 °C, 160 °C, 180 °C, and 200 °C, respectively.

**Figure 7:**
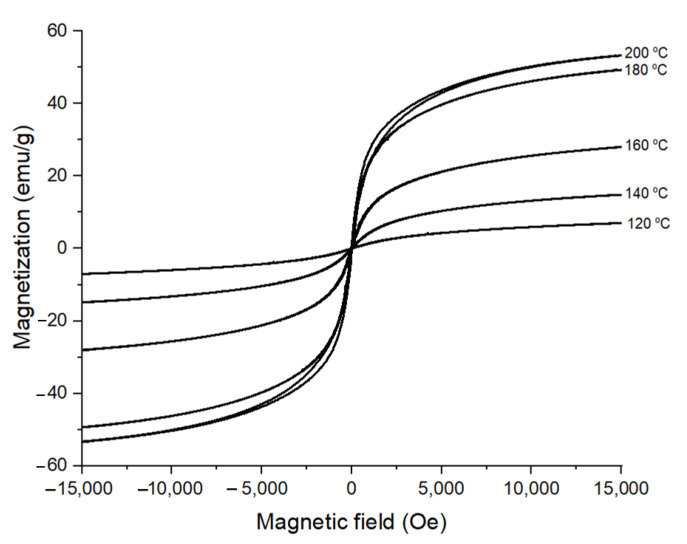
Room temperature M vs. H dependence of cobalt ferrite nanoparticles synthesized at 16 h in the presence of 1 M oleic acid for different reaction temperatures.

The magnetic properties of the CoFe_2_O_4_ nanoparticles prepared for different reaction temperatures with 1 M oleic acid are presented in Figure 7. It can be observed that, with the increase of reaction temperature, the magnetization saturation of the CoFe_2_O_4_ nanoparticles increased because the crystallinity of CoFe_2_O_4_ nanoparticles also increased. The magnetization value of the CoFe_2_O_4_ nanoparticles at 15,000 Oe of magnetic field increased from 7.03 emu/g to 14.85 emu/g, 27.98 emu/g, 49.35 emu/g, and, finally, 53.3 emu/g as the reaction temperature increased from 120 °C to 140 °C, 160 °C, 180 °C, and 200 °C, respectively. The magnetization value of the CoFe_2_O_4_ nanoparticles did not increase significantly for the temperature increase from 180 °C to 200 °C because the crystallinity was already developed enough for the temperature range. The CoFe_2_O_4_ nanoparticles prepared for the reaction temperature range of 120 °C–180 °C showed superparamagnetic behavior, while the sample prepared at 200 °C showed the ferromagnetic behavior. The transition in magnetic property from superparamagnetic to ferromagnetic behavior at 200 °C is believed to be coming from the increase of particle size [38,39,40]. As the particle size decreased below the critical size, magnetization can randomly flip the direction under the influence of temperature, causing the residual magnetization to be 0 [41]. These results were in good agreement with previously reported values of 6–10 nm for the critical particle size of CoFe_2_O_4_ nanoparticles [5,18].

### 3.3. Effect of Oleic Acid Concentration on the Morphology of CoFe_2_O_4_ Nanoparticles

**Figure 8:**
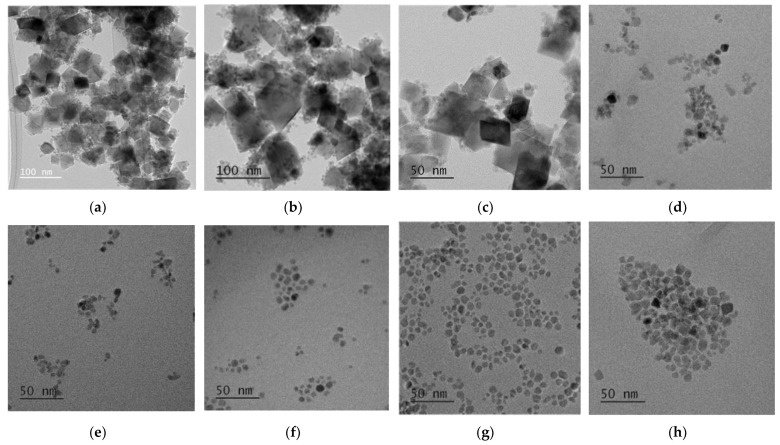
TEM results of cobalt ferrite nanoparticles synthesized at 180 °C for 16 h with different oleic acid concentrations of 0 M (**a**), 0.025 M (**b**), 0.05 M (**c**), 0.1 M (**d**), 0.15 M (**e**), 0.5 M (**f**), 1 M (**g**), and 1.5 M (**h**).

**Figure 9:**
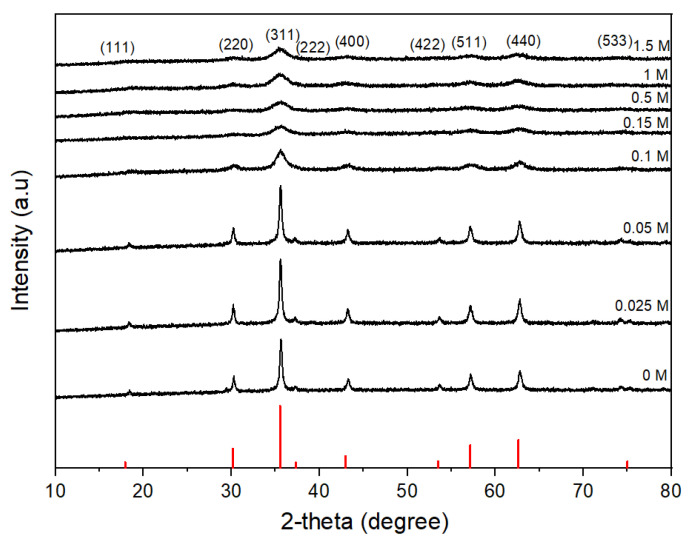
XRD patterns of cobalt ferrite nanoparticles synthesized at 180 °C, 16 h with different oleic acid concentrations.

**Figure 10:**
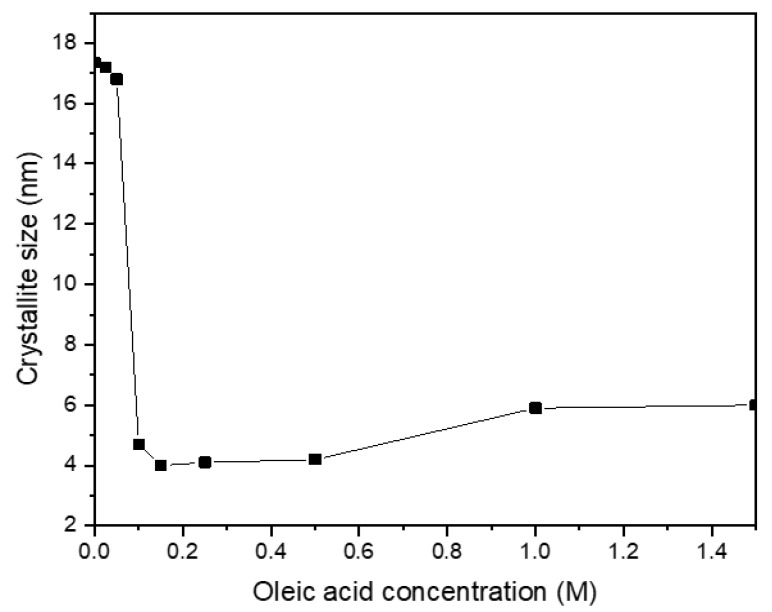
The average crystallite size as a function of oleic acid concentration calculated by using the Scherrer formula.

Figure 8 shows the TEM images of the CoFe_2_O_4_ nanoparticles prepared with different oleic acid concentrations (0 M–1.5 M) at 180 °C for 16 h. There were agglomerations between CoFe_2_O_4_ nanoparticles prepared without or with low oleic acid concentrations of 0.025 M and 0.05 M (Figure 8a–c). In these conditions, the large-sized CoFe_2_O_4_ nanoparticles of cubic shape were formed, and it was not easy to measure their independent sizes exactly because many particles were agglomerated together. For the oleic acid concentration of 0.1 M, most of the nanoparticles had the spherical shape, but a few had the large, cubic shape (Figure 8d). For the oleic acid concentration higher than 0.15 M, no cubic-shaped nanoparticle was observed. The average particle sizes of the CoFe_2_O_4_ nanoparticles prepared with oleic acid concentrations of 0.15 M, 0.5 M, 1 M, and 1.5 M were 6 nm, 6.3 nm, 7 nm, and 7.8 nm, respectively. The average particle size did not increase significantly when the oleic acid concentration was higher than 0.1 M. Figure 9 shows the XRD patterns of the CoFe_2_O_4_ nanoparticles prepared with different oleic acid concentrations (0 M–1.5 M) at 180 °C for 16 h. The cubic spinel structure of the CoFe_2_O_4_ nanoparticles was confirmed for all oleic acid concentrations (JCPDS card no. 22-1086). For the oleic acid concentrations from 0 M to 0.05 M, clear peaks including (220), (311), (400), (422), (511), (440), and (533) were presented with high intensity and the peaks of (111) and (222) were also observed with low intensity. For the oleic acid concentration of 0.1 M, all the diffraction peaks became broader and weaker with the increase of oleic acid concentration, but they became almost unchanged for the oleic acid concentration higher than 0.15 M. This can be explained by the decrease of CoFe_2_O_4_ crystallite size when the oleic acid concentration increased from 0 to 0.1 M. The average crystallite sizes with different oleic acid concentrations were found using the Scherrer formula, as shown in Figure 10. When the oleic acid concentration increased from 0.05 M to 0.1 M, the average crystallite size decreased abruptly but became almost constant for the oleic acid concentration higher than 0.1 M because the surface of the CoFe_2_O_4_ nanoparticles was almost fully covered by oleic acid and the diffusion of nanoparticle precursors from solution to CoFe_2_O_4_ nanoparticles was hindered and further growth of nanoparticles was inhibited.

**Figure 11:**
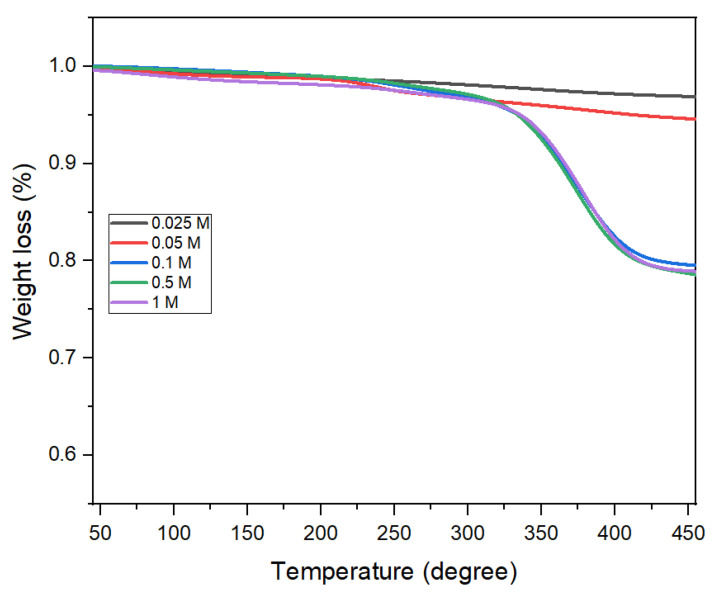
TGA results of cobalt ferrite nanoparticles synthesized at 180 °C, 16 h with different oleic acid concentrations.

Figure 11 shows the TGA results of samples to measure the amount of oleic acid adsorbed on the surface of the CoFe_2_O_4_ nanoparticles prepared with different oleic acid concentrations. The weight loss in the temperature range of 200–450 °C can be associated with the removal of oleic acid covering the surface of the CoFe_2_O_4_ nanoparticles (the boiling point of oleic acid is 360 °C). For the oleic acid concentration above 0.1 M, the weight loss was about 21% regardless of oleic acid concentration. The numbers of oleic acid ligands per unit surface area of the CoFe_2_O_4_ nanoparticles were 2.920 × 10^14^, 3.004 × 10^14,^ and 3.022 × 10^14^ with the oleic acid concentrations of 0.1 M, 0.5 M, and 1 M, respectively. These values did not change significantly for the oleic acid conditions here, which indicated that the CoFe_2_O_4_ nanoparticle surface was already fully adsorbed by the oleic acid at 0.1 M and no more adsorption was achieved for the oleic acid concentration higher than 0.1 M.

**Figure 12:**
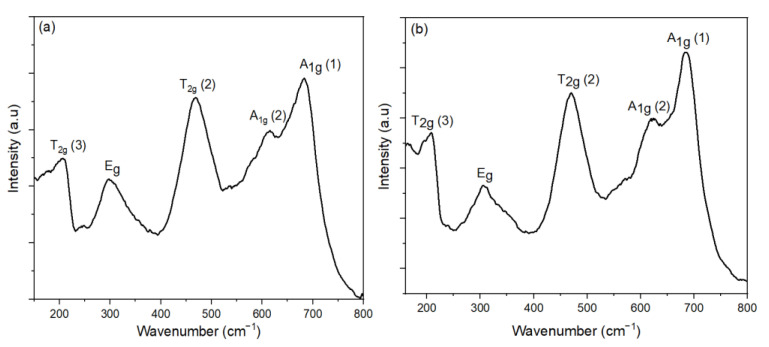

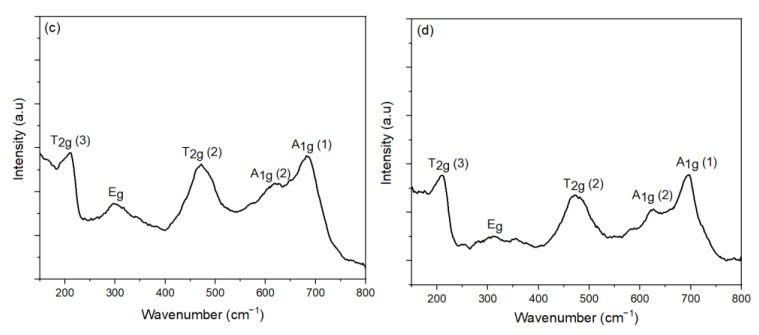
Raman spectra of the sample prepared with (**a**) 0 M, (**b**) 0.05 M, (**c**) 0.1 M, and (**d**) 1 M oleic acid concentration.

Raman spectroscopy was used to determine the degree of cation distribution (δ) for samples prepared with different oleic acid concentrations at 180 °C, 16 h, as shown in Figure 12. The Raman spectra showed the peaks at T_2g_ (3), E_g_, T_2g_ (2), A_1g_ (2), and A_1g_ (1) modes, which means that the CoFe_2_O_4_ nanoparticles of the spinel structure were synthesized. These bands, assigned as A_1g_ (1) and A_1g_ (2) modes, demonstrated the stretching vibrations of Fe–O and Co–O bonds, respectively, in the tetrahedral sites. The T_2g_ and E_g_ Raman modes demonstrated the vibration of the spinel structure. The Co contents in the tetrahedral site of the CoFe_2_O_4_ nanoparticles for oleic acid concentrations of 0 M, 0.05 M, 0.1 M, and 1 M were 0.303, 0.312, 0.31, and 0.32, respectively. Therefore, the oleic acid concentration had no significant influence on the cation distribution factor. The CoFe_2_O_4_ nanoparticles prepared with 1 M oleic acid concentration had the formula of (Co_0.32_Fe_0.68_)(Co_0.68_Fe_1.32_)O_4_ and δ here was 0.32.

**Figure 13:**
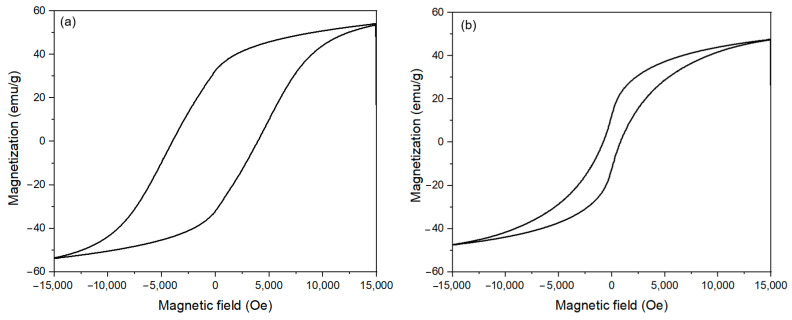

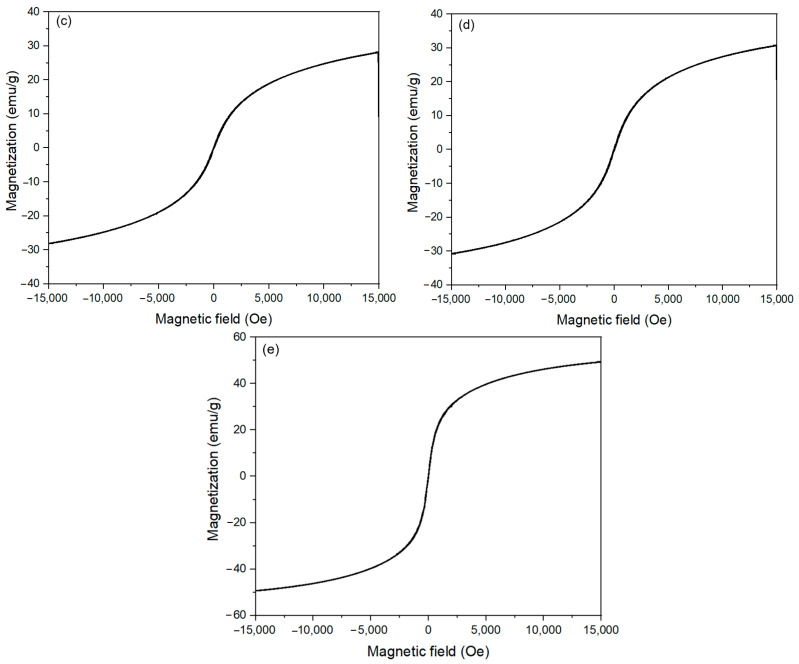
Room temperature M vs. H dependence of CoFe_2_O_4_ nanoparticles synthesized with different oleic acid concentrations, 0 M (**a**), 0.05 M (**b**), 0.1 M (**c**), 0.15 M (**d**), 1 M (**e**).

Magnetic properties of the CoFe_2_O_4_ nanoparticles prepared with different oleic acid concentrations were investigated by VSM measurement (Figure 13). The CoFe_2_O_4_ nanoparticles prepared with oleic acid concentrations lower than 0.05 M exhibited ferromagnetic behaviors, while the samples prepared with the oleic acid concentrations higher than 0.1 M showed the superparamagnetic behaviors. The change in magnetic behavior could be attributed to the decrease in particle size from multi-domain to single-domain structure when the oleic acid concentration reached a critical value of 0.1 M [37,42]. The formation of the single domain is detrimental to the energy, and, therefore, if thermal energy exceeds the magnetic anisotropy barrier, the residual magnetization becomes zero. The CoFe_2_O_4_ nanoparticles presented the superparamagnetic behavior with the particle size below the critical value [2]. The magnetic parameters such as saturation magnetization (M_S_) and coercivity (H_C_) from the hysteresis loops are listed in Table 3. The CoFe_2_O_4_ nanoparticles prepared with oleic acid showed lower magnetization saturation (M_S_) than the uncoated CoFe_2_O_4_ nanoparticles prepared at the same temperature. This was due to the effect of oleic acid coating where each particle was separated from its neighbors, leading to the decrease of magnetostatic coupling between the particles [43]. The values of coercivity decreased as the oleic acid concentration increased. Coercivity depends on many factors such as surface effect, defects, strains, non-magnetic atoms, and strains in the material [44]. Thus, the decrease in coercivity was explained by interfacial defect and the decrease in agglomeration also led to the smaller coercivity [45]. The increase in the magnetic value of the samples prepared with 1 M oleic acid concentration was due to the increase in particle size.

## 4. Conclusions

In this study, the CoFe_2_O_4_ magnetic nanoparticles were successfully synthesized by a solvothermal method with oleic acid as a surfactant. The effects of process variables such as reaction time, reaction temperature, and oleic acid concentration on the properties of CoFe_2_O_4_ nanoparticles were investigated. The oleic acid concentration played an important role in controlling the morphology and properties of the CoFe_2_O_4_ nanoparticles. A layer of oleic acid was coated on the surface of the CoFe_2_O_4_ nanoparticles immediately after 1 h of reaction time. This coating hindered further mass transfer of precursors from solution to nanoparticles, resulting in a negligible change in particle size with the increase of reaction time. The large-sized ferromagnetic CoFe_2_O_4_ nanoparticles with high magnetization were synthesized without or with low oleic acid concentrations. With the critical oleic acid concentration of 0.1 M, the small-sized, well-separated CoFe_2_O_4_ nanoparticles with superparamagnetic behavior were synthesized. A saturated layer of oleic acid was adsorbed on the surface of the CoFe_2_O_4_ nanoparticles when the oleic acid concentration reached a critical concentration of 0.1 M. This study will help prepare CoFe_2_O_4_ nanoparticles of high quality and also improve the performance of magnetic CoFe_2_O_4_ nanoparticles in many applications such as bio-separation, magnetic resonance imaging, biosensors, drug delivery, magnetic hyperthermia, etc.

## Figures and Tables

**Table 1:** EDS for % elemental composition of CoFe_2_O_4_ nanoparticles prepared at 180 °C, 16 h.

Element	Weight (%)	Atomic (%)
O	37.93	68.46
Fe	41.3	21.36
Co	20.77	10.18
Total	100	100

**Table 2:** List of peaks from FT-IR results.

Peak Number	Wavelength (cm^−1^)	Functional Group
1	3368.55	O–H
2	2919.52	CH_2_–
3	2850	CH_2_–
4	1530.4	COO–
5	1406.1	COO–
6	585.2	Fe–O

**Table 3:** Saturation magnetization (M_S_) and coercivity (H_C_) of samples prepared with different oleic acid concentrations.

Samples	Ms (emu/g)	Hc (Oe)
0 M	54.08	4012.8
0.05 M	47.53	841.9
0.1 M	29	0
0.15 M	32	0
1 M	49	0

## Data Availability

The data which indicated in this study are available on request from the corresponding author.
